# The Importance of Monitoring Antigen-Specific Memory B Cells, and How ImmunoSpot Assays Are Suitable for This Task

**DOI:** 10.3390/cells14030223

**Published:** 2025-02-05

**Authors:** Greg A. Kirchenbaum, Graham Pawelec, Paul V. Lehmann

**Affiliations:** 1 Research and Development, Cellular Technology Ltd. (CTL), Shaker Heights, OH 44122, USA; paul.lehmann@immunospot.com; 2 Department of Immunology, University of Tübingen, D-72076 Tübingen, Germany; graham.pawelec@uni-tuebingen.de; 3 Health Sciences North Research Institute, Sudbury, ON P3E 2H3, Canada

**Keywords:** ELISPOT, FluoroSpot, immune monitoring, affinity maturation, Ig class, high throughput, SARS-CoV-2, cross reactivity

## Abstract

Determining an individual’s humoral immune reactivity to a pathogen, autoantigen, or environmental agent is traditionally accomplished through the assessment of specific antibody levels in blood. However, in many instances, titers of specific antibodies decline over time and thus do not faithfully reveal prior antigen exposure or establishment of immunological memory. To estimate an individual’s humoral immune competence, it is therefore necessary to assess functional B cell memory. Here, we describe novel B cell ELISPOT and FluoroSpot assays (collectively referred to as ImmunoSpot) that can be rapidly developed and validated to characterize the memory B cell (B_mem_) repertoire specific for any desired antigen ex vivo and at single-cell resolution. Moreover, multiplexed variants of the B cell FluoroSpot assay enable high-throughput testing of antigen-specific B cells secreting distinct antibody classes and/or IgG subclasses, with minimal cell material requirements. B cell ImmunoSpot assays also enable measurement of affinity distributions within the antigen-specific B_mem_ compartment and permit cross-reactivity measurements that can provide insights into B_mem_ established against future pathogen variants. Collectively, the ImmunoSpot^®^ system presented here is highly reproducible, and can be readily validated for regulated tests. The newly gained ability to monitor the antigen-specific B_mem_ compartment should catalyze a more comprehensive understanding of humoral immunity in health and disease.

**Foreword**

Substantial progress has been made in minimalistic murine models towards the understanding of the cellular basis of B cell responses (Reviewed in [1,2]). In particular, it has become clear that the differentiation of B cells into antibody-secreting plasma cells (PCs), and memory B cells (B_mem_) underlies different affinity-based instructive lineage commitment pathways [3,4,5,6,7]. Subsequently, serum antibodies, commonly studied in humans, reflect PC generation, but only provide indirect insights—if any at all—into the development of the B_mem_ compartment. While the former is critical for the host’s ability to prevent (re-)infection (constituting the first wall of humoral defense), the latter is called upon to confer immune protection when the first wall fails. Hypotheses can be formulated based on these minimalistic murine models as to how the human B cell system will behave in complex infectious settings. However, such issues have not been tested experimentally in humans, mostly because of the lack of suitable B_mem_ detection systems. In this editorial, we outline the questions to be addressed to fill this gap and share our recent advances in reviving the B cell ImmunoSpot technology that might facilitate progress in this field.

## 1. Introduction

Assessments of whether an individual possesses protective immunity to a specific pathogen after natural infection, or following vaccination, are crucial surrogates (“biomarkers”) for predicting clinical protection against exposure or re-exposure to that pathogen [8]. Additionally, the detection of self-reactive antibodies plays a critical role in the diagnosis of autoimmune diseases [9] or allergies [10]. Antibodies also contribute to organ transplant rejection [11] and anti-tumor immunity [12]. In each of these instances, routine clinical diagnostic assessments are commonly based solely on the presence of measurable antibody reactivity in blood (plasma or serum). Of note, assessments of antibody binding or neutralizing activity in blood yield nearly identical results when performed using either plasma or serum samples [13,14], which principally differ only in the presence or absence of clotting factors. Here, we argue that to acquire a more comprehensive assessment of an individual’s humoral immune status, it is mandatory to measure antigen-specific B_mem_ themselves. This is because antibody measurements performed using plasma or serum reflect only one transient arm of the humoral defense (the “first wall”, as defined below), and frequently provide either incomplete or even false-negative information on the other, i.e., the antigen-specific B_mem_ compartment (the “second wall”), that in large part has gone unstudied so far.

There are multiple reasons why B_mem_ assays are currently excluded from standard immune diagnostics, the most likely of which is that the quantification of antibody titers, quite straightforward to measure, has been assumed to accurately reflect on the antigen-specific B_mem_ compartment as well. This rendered it apparently unnecessary to pursue complicated and expensive functional cellular assays, also perceived as challenging to standardize, which are needed to directly examine the antigen-specific B_mem_ themselves. However, recently it has become more apparent that terminally differentiated antibody-secreting PCs and long-lived B_mem_, which retain the capacity to rapidly respond to a future pathogen challenge, do not arise in a fixed ratio to each other [15], and consequently that circulating antibody levels (in plasma/serum) and B_mem_ frequencies are frequently discordant [16]. Therefore, assessing only antibodies originating from PCs cannot provide a full picture of an individual’s humoral immune competence. Clarity in the analysis of B cell lineage differentiation pathways has resulted in the acceptance of an alternative paradigm (reviewed in [17]), which will be discussed in the following.

## 2. Memory Cell or Plasma Cell Development?—B Cell Fate Decisions

**Figure 1:**
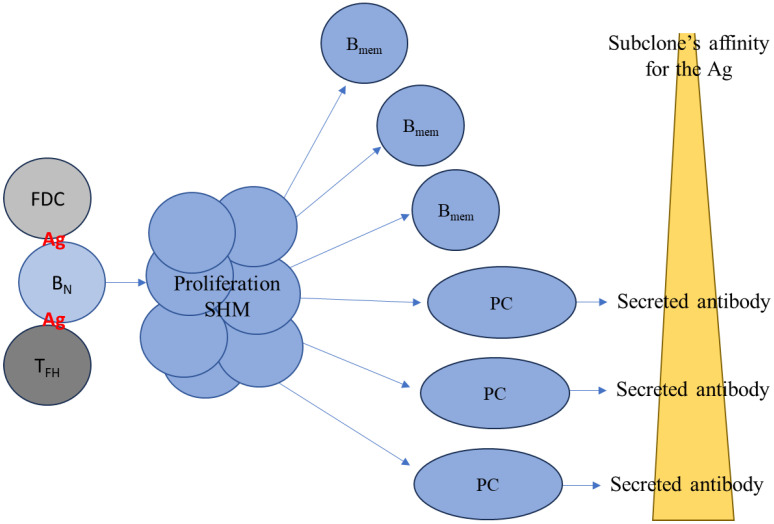
Affinity determines the differentiation pathways of plasma and memory B cells. Most commonly in a lymph node, a naïve B cell (B_N_) encounters the homotypic antigen (Ag) for which its BCR has significant affinity (e.g., >Kd^−5^) and is activated following cognate interactions with follicular T helper cells (T_FH_) and follicular dendritic cells (FDCs). The B cell enters a germinal center (GC) within the lymph node, where it clonally expands, undergoes Ig class switching, and acquires somatic hypermutation (SHM) of its BCR. As SHM is a random process, the progeny of the original B_N_ cell express BCRs with a broad spectrum of affinities for the homotypic antigen. Daughter cells (subclones) expressing high-affinity BCRs are retained in the GC, and after multiple rounds of cell division, SHM, and positive selection, they eventually differentiate into plasma cells (PCs). Progeny with BCRs of lower affinity for the homotypic antigen exit the lymph node as memory B cells (B_mem_). However, as a consequence, B_mem_ with low affinity for the homotypic antigen may be endowed with an increased affinity for a heterotypic antigen variant to be encountered in the future.

**Figure 2:**
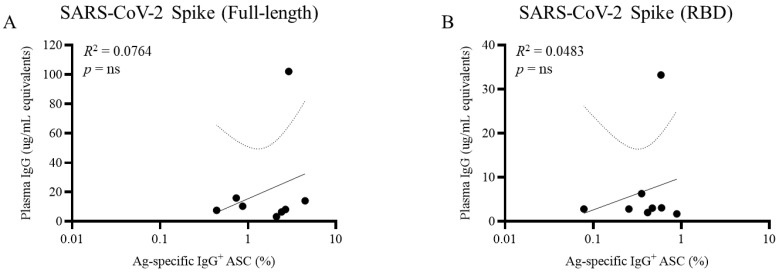
Discordance between plasma antibody levels and frequency of memory B cells (B_mem_) against the ancestral SARS-CoV-2 S antigen (Wuhan-Hu-1). COVID-19 mRNA-vaccinated donors (*n* = 8) with no history of prior SARS-CoV-2 infection were assessed ca. 6 months following completion of the initial prime-boost vaccination regimen for circulating antibody reactivity in plasma against (**A**) full-length Spike (S) antigen or (**B**) receptor binding domain (RBD) by ELISA (y-axis) or S antigen-specific B_mem_-derived IgG^+^ antibody-secreting cells (ASC) following polyclonal stimulation of cryopreserved PBMC by ImmunoSpot (x-axis). Each symbol represents one individual. Note that certain individuals possess a high frequency of B_mem_-derived IgG^+^ ASC but low or undetectable levels of S antigen-specific IgG reactivity in the corresponding plasma sample. Correlation analysis between plasma antibody levels and frequency of S antigen-specific B_mem_-derived ASCs was performed according to the methods described previously [16].

Traditionally, antigen-specific B cell responses lead to the formation of germinal centers (GCs) within the spleen and/or draining lymph node(s) (LN). It is now widely accepted that the generation of long-lived B_mem_ and PCs in GCs is controlled by the affinity of the individual differentiating GC B cell’s antigen receptor (Figure 1 and [2,18]). As B cells proliferate and their antigen receptors undergo somatic hypermutation (SHM) within the GC, progeny arise bearing receptors with different affinities for the eliciting antigen (referred to as the homotypic antigen). Because the acquisition of SHMs is largely random, some of the mutated B cell receptors (BCRs) will, by chance, have acquired an increased affinity for the homotypic antigen, but most will not. (The significance of the latter is, however, that they may have increased affinity for related heterotypic antigenic variants: see below). Differentiating GC B cells endowed with receptors with an increased affinity for the antigen will preferentially receive stimulatory cues (provided by follicular helper T cells) instructing them to undergo additional rounds of proliferation with accompanying SHM of their BCR. Following multiple waves of successive positive selection, the GC B cells which have attained the highest affinity for the homotypic antigen will differentiate into PCs capable of secreting large quantities of affinity-matured antibodies. In contrast, GC B cells that underwent positive selection but still express lower affinity BCR for the homotypic antigen will exit the GC and enter into the long-lived B_mem_ compartment. Thus, PCs and B_mem_ differentiate along different pathways controlled by BCR affinity-based selection for the homotypic antigen. Consequently, neither the frequency nor the affinity distribution of the resulting PC and B_mem_ repertoires are mirror images of each other, and neither are the fates and lifespans of these two cell types [15,19]. Hence the abundance of specific antibodies in plasma/serum (and other bodily fluids) and the frequency of antigen-specific B_mem_ are not inherently linked [16]. Therefore, while both B_mem_ and PCs originate from the same progenitor B cell pool, they possess fundamentally different roles for mediating humoral immune defense [17].

## 3. Circulating Antibodies Represent the First Wall of Humoral Defense, Memory B Cells the Second

The PCs that arise during an immune response can be long-lived and are capable of secreting large quantities of specific antibodies for many years (potentially decades) [19,20]. However, PCs must take up residence in specialized environmental niches, such as within the bone marrow, to achieve this extended survival [21]. Recent data suggest that PC lifespans are heterogeneous and fall on a continuum, with only a small fraction surviving for >60 days [22]. The antibody-secreting cell (ASC) turnover is set, therefore, by intrinsic lifespan limits, with steady-state population dynamics governed by niche vacancy rather than merely by displacement. This dynamic affects the antibody titer measured in plasma/serum at any particular time. This is because secreted antibody molecules have short half-lives in vivo and subsequently, the maintenance of circulating antibody titers in plasma/serum requires constant replenishment by PCs. The half-lives of IgG1, IgG2, and IgG4 in humans are 21–28 days, whereas for IgG3 it is only a week [23]. The half-lives of IgA and IgM are shorter at only 3–7 days [24,25] and IgE exhibits the shortest half-life of 2–3 days [26].

The specific antibodies in plasma/serum produced following an initial (“primary”) immune response aim not only at eliminating the antigen but also later on to prevent the re-entry and dissemination of the same (homotypic) antigen. Such pre-formed antibodies serve as an immediately deployable “first wall” of humoral immune defense. If such antibody titers decline, however, they fail to confer protection from (re-)infection. Crucially, even when protective antibody levels are maintained, or further boosted through additional vaccination(s) against the homotypic pathogen strain, the evolution of variants under pressure to evade neutralizing activity can still cause breakthrough infection(s) with heterotypic variants, as seen for example with circulating seasonal influenza isolates, or in the recent COVID-19 pandemic. Consequently, increasing antibody titers through the administration of booster vaccinations with the homotypic strain is not necessarily going to be effective at increasing protection against emerging heterotypic variants. However, when encountering the heterotype, the immune response will not necessarily have to “start from scratch” in generating an effective antibody response, because this is where the antigen-experienced and somatically-mutated B_mem_ repertoire acquires its critical importance. Inevitably, there will be BCRs present within the B_mem_ pool elicited by the homotypic antigen that were not selected for continued affinity maturation and terminal differentiation into PCs, yet at least a fraction of these BCRs will have an adequate, if not increased, affinity for a heterotypic variant. Moreover, such heterotype-specific B_mem_ is present at much higher frequencies than would otherwise exist in a naive B cell pool, and further, many of such B_mem_ would already have undergone Ig class-switch recombination (CSR). Hence, such heterotype-specific B_mem_ is poised for re-engagement into a secondary-type antibody response even at the first encounter with the heterotypic variant, enabling the development of more rapid and robust humoral immunity. The heterotype-specific B_mem_ can either rapidly differentiate into ASCs following an antigen-stimulated proliferative burst, or they can (re-)enter GCs in order to acquire additional somatic mutations that serve to refine their BCR affinity for the new variant antigen. In this way, B_mem_ provides the “second wall” of humoral host defense.

From the above considerations, it is clear that assessing antigen-specific antibodies in plasma/serum provides only a snapshot of the current status of humoral immunity and says little about actual B_mem_ and nothing at all about the ability of an individual to respond to renewed challenges by the same or a variant pathogen. In contrast, assessing the capacities of B_mem_ to recognize target antigens provides valuable information on the B cell system’s potential to engage in defense reactions in the future.

## 4. Accumulating Evidence Suggests That Circulating Antibody Titers and Frequencies of Peripheral Memory B Cells in Blood Yield Different Information About Humoral Immunity

We previously reported an extensive study comparing anti-SARS-CoV-2, EBV, and seasonal influenza antibody titers in plasma with B_mem_ frequencies in PBMC specific for the same antigens [16]. We believe this study to be the first of its kind, made possible by the recent development of the ImmunoSpot technique that enabled robust and reproducible identification and quantification of the relevant B_mem_. We found that many individuals with PCR-verified SARS-CoV-2 infections had essentially undetectable antibody reactivity in plasma, but nonetheless, some possessed highly increased frequencies of antigen-specific B_mem_. We have also observed this phenomenon in the case of the persistent herpesvirus human cytomegalovirus (HCMV) [27], with B_mem_ (and T cells) detectable in the absence (or near-absence) of specific antibody reactivity in blood. Thus, compared with antibody titers, assessment of B_mem_ may be more accurate for assessing prior antigen exposure, i.e., individuals who are essentially seronegative for a certain antigen may often possess antigen-specific B_mem_, sometimes even at high frequencies (e.g., see Figure 2 and [16]). The potential clinical relevance of these studies should not be overlooked. The disparity between seronegativity for HCMV despite clear evidence for HCMV antigen-specific B_mem_ (and T) cells in donors sourced from FDA-approved blood banks [27] suggests that routine serological testing of blood donors for HCMV, a potentially dangerous pathogen in immunosuppressed individuals, may not identify all infected donors.

Of the antigens that we tested in this regard, SARS-CoV-2 proteins were particularly informative because SARS-CoV-2 Spike (S) and Nucleocapsid (N) antigens were neoantigens before the emergence and spread of this virus, and B_mem_ specific for SARS-CoV-2 S or N antigens were essentially undetectable by ImmunoSpot assays in PBMC cryopreserved before the COVID-19 pandemic [16]. Nevertheless, despite the absence of B_mem_-derived IgG^+^ ASC reactivity against the SARS-CoV-2 N antigen, several of the corresponding plasma samples provided false-positive results when evaluated for N antigen-specific IgG via ELISA. However, for PBMC collected during the pandemic, the presence of antigen-specific B_mem_ provided 100% diagnostic specificity for subjects with PCR-confirmed SARS-CoV-2 infection (much more reliably than did seropositivity) [28]. Furthermore, SARS-CoV-2 N antigen-specific B_mem_ (a surrogate for prior SARS-CoV-2 infection) was detected in many PBMC samples collected in the post-COVID era and served to document the widespread of the SARS-CoV-2 virus, whereas the absence of N antigen-specific antibody reactivity in plasma samples frequently yielded a false-negative test result [29]. However, since antigen-primed B_mem_ can also reside in non-lymphoid tissues (reviewed in [30]), it is plausible, at least for some antigens, that the absence of detectable B_mem_ reactivity in PBMC would not exclude the existence of tissue-resident B_mem_ in the host. This remains an open question and challenge in the field of cellular immune monitoring and would necessitate a parallel assessment of tissue biopsies and paired PBMC samples originating from donors with confirmed antigen exposures. Nevertheless, the ImmunoSpot assay approach is ideally suited for such experimentation owing to the minimal cell material required to perform assays, and the exquisite sensitivity afforded through measuring individual ASCs at single-cell resolution.

The introduction of the SARS-CoV-2 virus into the human population also served as an unprecedented “real world” opportunity to study B_mem_ owing to the collection and cryopreservation of PBMC samples from individuals with defined exposure histories (e.g., infection vs. vaccination). Such an ideal scenario is extremely rare for basic research efforts because of the otherwise complex and asynchronous exposure histories of the general human population. Defining the basics of B_mem_ induction and their behavior upon subsequent antigen encounters in this context is fundamental for advancing the blossoming field of human B_mem_ research. Leveraging the many lessons learned in the context of SARS-CoV-2 will certainly continue to contribute to an overall improved understanding of B_mem_, with the ultimate goal of unveiling insights into more complex scenarios that are commonplace in human immunological research.

## 5. Development of Next Generation B Cell ImmunoSpot Assays for Essentially Any Antigen by Means of Affinity Coating

**Figure 3:**
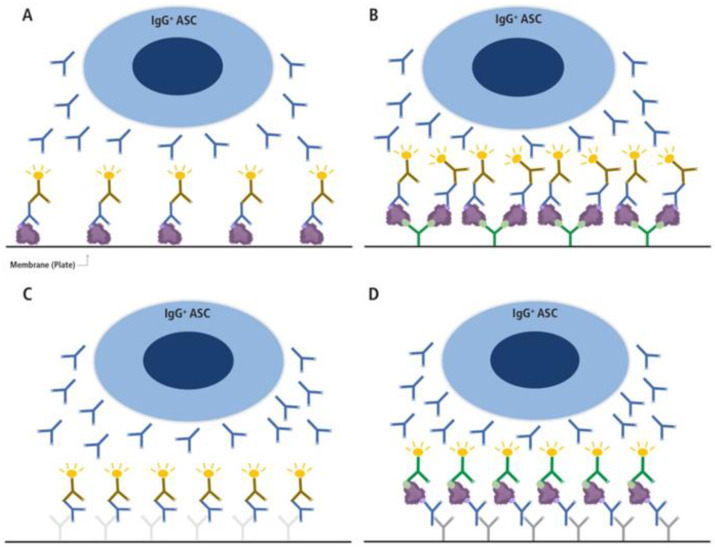
Different types of B cell ImmunoSpot assays. (**A**,**B**) direct antigen-specific assay variants, (**C**) the pan Ig detecting test, and (**D**) the inverted antigen-specific assay. In the direct antigen-specific assay variant (**A**), the antigen itself is coated onto the membrane directly, as has been done traditionally, whereas in variant (**B**), the antigen’s binding to the membrane is aided by high-affinity capture utilizing an affinity tag. For the latter, a His-tagged recombinant protein (depicted as a purple blob, and the His-tag epitope denoted in light green) is captured onto the membrane with high affinity via the plate-bound anti-His antibody (depicted in green) [31]. In A and B, only Ig produced by antigen-specific antibody-secreting cells (ASCs) with sufficient binding affinity will be retained on the lawn of antigen bound on the membrane (the ASC-derived antibodies are depicted in blue in all panels) and are visualized by adding an anti-human Ig detection antibody (in the example shown, anti-IgG, depicted in brown). In the pan Ig detecting assay (**C**), the Ig produced by ASC is captured by an anti-species antibody coated onto the membrane (e.g., a goat anti-human Igκ/λ, depicted in light grey), and the plate-bound human IgG is visualized using an anti-human IgG Fc-specific detection antibody (depicted in brown). In this assay variant, as with the inverted assay (**D**), secretory footprints generated by IgG-producing ASC are captured irrespective of their antigen-specificity. In the inverted assay (**D**), the membrane is coated with an anti-human IgG Fc-specific capture antibody (depicted in dark gray), and the soluble antigen (depicted as a purple blob, and the His-tag epitope denoted in light green) will only be captured by secretory footprints generated by antigen-specific IgG^+^ ASC. The membrane-bound antigen is detected in a subsequent step via a detection reagent in the example shown, and the His-tagged recombinant antigen is detected via an anti-His tag-specific detection antibody (depicted in green).

While there are compelling reasons for monitoring antigen-specific B_mem_, and while this goal (in principle at least) can be readily accomplished by ImmunoSpot, one might ask why immune monitoring rarely involves B_mem_ detection. The simplest reason is that the classic protocol involving direct coating of the membrane (Figure 3A) fails for most antigens. Having tested many antigens, we found that direct antigen coating to the assay membrane was either insufficient for detecting antigen-specific ASCs or at best resulted in faint barely detectable secretory footprints (“spots”) even when prohibitively high (and costly) antigen concentrations were used. To overcome this obstacle, and to facilitate a universal approach for achieving high-density antigen coating, we pioneered the technique of “affinity capture coating”; the membrane is first coated with an anti-affinity (His- or other) tag antibody, and then recombinant (His- or other) affinity-tagged antigen is added [31]. Thus, the first-generation approach that relied on weak, non-specific binding (primarily via hydrophobicity) of the antigen to the assay membrane can be replaced with specific, high-affinity binding that facilitates reliable and high-density antigen coating at greatly reduced antigen coating concentrations. As a result, ImmunoSpot assays can be developed for essentially any tagged antigen, as illustrated in Figure 3B. Furthermore, leveraging this innovation, variants of B cell ImmunoSpot can be developed to assess additional aspects of B_mem_ and ASC functional properties. These approaches are described in the following sections.

## 6. Detecting and Characterizing Memory B Cells by ImmunoSpot vs. Flow Cytometry

An established method for detecting antigen-specific B cells relies on labeling the cells with fluorescence-tagged antigens in order to quantify them by flow cytometry (FCM). A strength of this method is that it allows the antigen-specific B cells to be phenotyped for other surface markers at the same time, thus enabling the identification of antigen-binding B cell subsets and/or isolation for downstream applications (e.g., repertoire sequencing). However, FCM also has numerous disadvantages compared to ImmunoSpot, most notably the greater sensitivity of the latter: ImmunoSpot allows the identification of a single ASC in PBMC, easily down to 1 in 10^5^, and potentially much lower [32], which cannot be detected in bulk populations by FCM. However, as B_mem_ are resting lymphocytes that do not spontaneously secrete antibodies, an in vitro polyclonal stimulation culture is required to convert them into ASCs that can be detected in the ImmunoSpot assay. In contrast, plasmablasts that have been engaged in an immune response following antigen encounter in vivo can be detected by ImmunoSpot assays directly ex vivo. The latter represent precursors of PCs that appear transiently in blood during their migration from lymph nodes or other lymphoid tissues to the bone marrow or alternative niches where they may reside long-term. Not being part of the long-lived B_mem_ compartment, such plasmablasts are not discussed in greater detail in this communication. Importantly, only the cell numbers available for the ImmunoSpot assay itself define the lower limits of detecting rare ASCs provided that test samples are plated at ≤5 × 10^5^ PBMC per well—to avoid cell crowding and compromised detection of each cell’s secretory footprint—in one or more replicate wells [33].

Another advantage of ImmunoSpot over FCM is that substantially lower numbers of PBMC are required for ImmunoSpot-based identification of antigen-specific B cells [34]. With less than 5 × 10^6^ cryopreserved PBMC per antigen, the frequency of the antigen-specific B cells secreting all four Ig classes and IgG subclasses can be determined, plus the frequencies of antigen-specific B cells amongst all ASCs producing these antibody subtypes. Moreover, from a practical point of view, the level of technical skill required for accurate multiparametric FCM is far greater than that necessary for carrying out even 4-color B cell ImmunoSpot tests [34]. The existing protocols enable GLP-compliant high-throughput measurements allowing the convenient identification of the Ig class/subclass of antibody produced by the antigen-specific ASCs [32]. FCM, in contrast, does not reliably reveal the Ig class/subclass that will be produced by individual B cells because surface BCR expression can be highly variable; this is an underappreciated complexity of probe staining [35]. Moreover, in the case of IgG-secreting B cells, little if any BCR is present on the cell surface; this undermines the assessment of their antigen specificity and subclass use by traditional surface staining approaches [36,37,38]. Consequently, fixation and intracellular staining are required to define IgG subclass usage, a procedure that results in substantial cell loss in the sample. Finally, the B cell ImmunoSpot assay is suitable for high-throughput investigation, with the additional advantage that even multi-color ImmunoSpot^®^ analysis can be fully automated.

## 7. Identification of Ig Class/Subclass Production by Antigen-Specific Memory B Cells

Each of the different Ig classes and subclasses has distinct effector functions making unique contributions to host defense [39]. ImmunoSpot assays permit the determination of the frequency of antigen-specific B cells expressing these classes/subclasses at single-cell resolution, and hence their ratios relative to each other within the antigen-specific repertoire. Following their initial activation, IgM^+^ naive B cells can differentiate into effector cells (PCs) that have undergone CSR, and into B_mem_ [15,17]. CSR is irreversible and involves excising the exons of the Igμ heavy chain gene necessary for IgM expression. This is followed by the rearrangement of the upstream variable region genes (VDJ, that are responsible for defining the BCR or secreted antibodies’ antigen specificity), with downstream exons specifying the different Ig classes or IgG subclasses [40]. CSR is influenced by several factors, primarily the type of “help” provided by CD4^+^ T cells. Optimal Ig class deployment on infection or after vaccination is crucial for adequate host defense in the absence of significant immunopathology (reviewed in [41]). Upon subsequent rechallenge with the homo- (or hetero-) typic antigen, B_mem_ predominately differentiates into PCs producing the same Ig class/subclass expressed by the parental B_mem_. Because of this, ImmunoSpot assays permit prediction of the class/subclass usage of antibodies that will be secreted following a future antigen (re-)exposure. Knowledge of the full spectrum of Ig classes/subclasses that the pool of antigen-specific B_mem_ will produce after the next antigen encounter is thus of paramount importance for forecasting the success of future humoral immunity to that antigen. One important question that remains an area of investigation is the utility of B cell ImmunoSpot assays for measuring frequencies of antigen-specific IgM^+^ B_mem_, which have received increased attention in recent years (reviewed in [42]) because TLR-driven (R848+rIL-2) polyclonal stimulation also drives terminal differentiation of IgM^+^ naïve B cells present amongst the donor PBMC [43]. Nevertheless, while the majority of antigen-specific reactivity in PBMC resides in the IgG^+^ ASC compartment, we routinely perform multiplexed B cell ImmunoSpot^®^ assays, without any appreciable loss in sensitivity, that enable simultaneous assessment of ASC producing any of the four major Ig classes (IgM/IgA/IgG/IgE) or IgG subclasses (IgG1/IgG2/IgG3/IgG4) [28,32] to enable comprehensive detection of all antigen-specific ASCs.

Determining the frequency of antigen-specific B cells can be accomplished by counting the secretory footprints as specific spot-forming units (SFUs). These can be expressed simply as SFU per cells plated per well, or, more informatively as the frequency of antigen-specific ASCs producing a particular Ig class or subclass within the totality of all ASCs secreting that Ig class/subclass. For this, care must be taken to avoid crowding of SFU, as well as an ELISA effect, both of which may result in underestimation of the antigen-specific ASC frequency [16]. We have systematically evaluated these variables, concluding that a range of cell numbers plated per well can be established where SFU numbers are directly proportional to the number of cells plated [34]. Outside of this range, which varies according to the type of assay and morphology of the SFU arising, the relationship may break down at around 100–200 SFU per well. This can represent a significant hurdle when accurately estimating frequencies of antigen-specific B_mem_, particularly in view of our earlier data showing enormous inter-individual variation in the frequencies of these cells which may even span orders of magnitude for the same antigen [16]. Furthermore, considerable intra-individual variation is also present, with the frequency of antigen-specific B_mem_ for different antigens having a similarly broad distribution [33]. Adding yet more complexity, even the number of ASCs producing a certain Ig class or subclass, regardless of antigen specificity, also exhibits a high degree of inter-individual variation. A solution to this problem is to plate serial dilutions of cells to identify the linear range of SFU counts and to estimate frequencies only within this range [32,33]. To render this approach feasible for use in high-throughput workflows, software for automatically calculating frequencies from this type of serial dilution experiment has also been developed [32,44]. In concert with the front-end planning module incorporated into the ImmunoSpot^®^ software—defining the samples’ identity, cell input(s) tested, and assay-specific counting parameters to be deployed following automated robotic image acquisition—the data analysis has been fully automated.

## 8. Quantifying the Affinity Distribution of the Antigen-Specific Memory B Cell Pool

As noted above, SHM of the BCR results in the generation of a diverse B_mem_ pool from the population of B cells that previously entered the GC reaction [45]. This pool of B_mem_ possesses a range of BCR affinities for the reencountered antigen, whether homo- or heterotypic, and may include those with a higher affinity for the heterotype [46].

The equilibrium dissociation constant (K_D_) between the antibody and its cognate antigen is referred to as the affinity of the antibody and defines the amount of soluble antibody required to attain 50% of the maximal antigen binding capacity. Because the forces mediating antibody binding to the antigen are noncovalent, the attachment is reversible, resulting in a constant “on-off” flickering of the paratope-epitope association following a second-order biochemical reaction. The affinity of antibodies elicited over the course of an immune response can range from low (e.g., K_D_ < 10^−5^ M) to high (e.g., K_D_ > 10^−10^). Thus, a difference of >10^5^-fold in the amount of antibody required to achieve the same degree of epitope coverage would be required for Ig produced by a low affinity relative to a high-affinity B cell to mediate the same effector function(s). Hence, “specific” antigen binding will be measurable for a plethora of antibodies present in blood (plasma/serum) provided they are at a sufficiently high concentration, but 10^5^-fold more molecules of a low-affinity antibody relative to a high-affinity antibody would be required to achieve the same level of biological activity. It is, therefore, crucial to establish the distribution of antibody affinities in the antigen-specific B_mem_ repertoire for any given antigen, rather than simply enumerating the frequency of such antigen-specific ASCs, because this is what determines their actual contribution to protective immunity. As classical methods for antibody affinity measurements require the generation of monoclonal antibodies and their evaluation individually, this process is impractical for high-throughput immune monitoring of multiple antigen-specific B_mem_ repertoires in sizeable human cohorts.

In marked contrast, affinity distribution data on the antigen-specific B cell repertoire are easily obtainable using ImmunoSpot approaches [47]. Briefly, as an example, if the affinity distribution of antibodies from IgG1-producing B_mem_ is to be established, the first step is to determine the frequency of antigen-specific IgG1^+^ ASCs using the single well serial dilution method [34]. Additionally, to calculate the frequency of antigen-specific IgG1^+^ ASCs amongst all IgG1^+^ ASCs, it is necessary to measure all IgG1^+^ ASCs irrespective of their specificity in a pan Ig test (Figure 3C). The second step is to perform an “inverted assay” using a replicate vial of cryopreserved cells whereby the plate is coated with an anti-IgG1 capture reagent, and the pre-determined input for the test cell population is added (Figure 3D). The number of cells to be added to yield secretory footprints at the upper threshold of the linear range would be determined in the preceding experiment using a serial dilution approach and a “saturating” concentration of the detection probe. This optimal cell input can be referred to as the “Goldilocks number” and aims to achieve around 50 SFU per well. Because every SFU originates from a single ASC, at 50 SFU per well, the secretory footprints of 50 ASCs are directly assessed, and this number can be increased proportionally by seeding cell material into additional replicate wells; examining ~600 secretory footprints (in 12 replicate wells) at each concentration of the antigen probe provides a substantial representative sample size for the antigen-specific B cell repertoire in each individual at the timepoint of sample collection. This technique necessitates adding the antigen probe in titrated concentrations. Secretory footprints of all the antigen-specific ASCs in the population, both high and low affinity, are detected at the highest (“saturating”) antigen probe concentration. With ever-decreasing concentrations of antigen, ASCs producing ever higher affinity antibodies remain detectable. The affinity range of the ASC repertoire is assessed by plotting SFU lost at each decreasing antigen concentration, which reveals the percentage of antigen-specific ASCs with affinities less than the minimal threshold.

Following these protocols thus facilitates an assessment of the affinity distribution within the antigen-specific ASC repertoire. Moreover, B cell ImmunoSpot assays can illuminate affinity distributions by at least two additional approaches [47]. First, by determining the morphology of the SFUs detected when antigen is directly coated onto the membrane, as described in Figure 3A,B. Secretory footprints originating from ASCs producing higher affinity antibodies appear as smaller, sharper, and more dense spots, whereas ASCs producing lower affinity antibodies yield secretory footprints that are larger, fainter, and more diffuse. Scatter plots depicting the size and density of the spots can be processed to extract such information. Second, coating the membrane with progressively lower (sub-optimal) concentrations of antigen and studying alterations in SFU counts and their morphology, respectively, also allows the affinity distribution of the antigen-specific ASC repertoire to be evaluated.

## 9. Cross-Reactivity of Memory B Cells at the Single Cell Level

As discussed above, B_mem_ acts as a “second wall” of defense against heterotypic antigens, particularly those derived from viruses that have evolved variants no longer recognized by the neutralizing antibodies that were previously generated against the original homotypic strain. As B cell differentiation into PCs vs. B_mem_ is affinity-driven, low-affinity heterotype-specific B_mem_ can be induced in the absence of heterotype-specific antibodies (PCs). Therefore, assessing only the presence of circulating antibodies in blood (plasma/serum) provides no data from which one could predict the existence of heterotype-reactive B_mem_ that can contribute to enhanced protection, whereas investigations of B_mem_ are highly informative in this regard.

The presence of cross-reactivity within the B_mem_ compartment can be investigated by ImmunoSpot using two distinct testing strategies. One strategy, which is applicable when a panel of heterotype(s) has been defined in advance, involves serial dilution of the test sample in ImmunoSpot assays (direct antigen coating or inverted, see Figure 3) performed in parallel. In this way, the homotypic and heterotypic ASC frequencies can be directly compared to provide an assessment of existing cross-reactivity (albeit at low resolution). The second strategy offers a higher resolution assessment of cross-reactivity, but importantly first requires that the number of input cells per well necessary to achieve the “Goldilocks” SFU count for the homotypic antigen (about 50 SFU per well, see above) is already known. In the simpler version, a single-color inverted B cell assay (Figure 3D) is performed using the Goldilocks cell input number. Seeding replicate wells at the predetermined Goldilocks cell input (e.g., 12 wells, yielding ~600 individual homotypic antigen-specific SFU), the cumulative number of SFU revealed using the homotypic antigen probe can be directly compared with the number of SFU detected using the heterotypic antigen(s). Furthermore, titrating the concentrations of the homo- and heterotypic antigen probes enables assessment of the affinity distribution of the heterotype-specific ASC repertoire compared to that reactive with the homotype.

Lastly, and building on the “Goldilocks” cell input tactic, a more elegant approach involves assessing B cell cross-reactivity through competitive co-labeling of secretory footprints using both homotypic and heterotypic antigen probes simultaneously. As before, an inverted assay is employed using PBMC (or an alternative cell suspension) plated at the predetermined Goldilocks cell input per well for the homotypic antigen. A “tag 1”-labeled homotypic antigen (for example, using a FLAG-tagged antigen) and a “tag 2”-labeled heterotypic antigen (for example, His-tagged antigen) are added simultaneously at equimolar concentrations. Secretory footprints that are co-labeled with both antigen probes (when used at equimolar concentrations) originate from ASCs that are unequivocally cross-reactive. Additionally, this approach allows for the identification of ASC-derived secretory footprints that are solely reactive with either the homotypic or heterotypic antigen probe. Moreover, the affinity distribution of ASCs for either the homotypic or heterotypic antigen probe can be evaluated by titrating the concentration of one probe while maintaining the other probe at a sufficiently high, but importantly non-saturating, concentration.

## 10. Concluding Remarks

The intent of this editorial is to draw attention to the thus-far little-exploited potential of B_mem_ investigations to enhance immune diagnostics. Despite the fact that ELISPOT assays were in fact first developed for quantifying antigen-specific ASCs [48,49], nonetheless, the T cell ELISPOT assay developed much later [50] has instead become the most widely applied. Modifications to the original T cell ELISPOT protocol allowed the assay to reliably reveal the secretory footprints of individual T cell responses to antigen [51] due to our introduction of the PVDF membrane with its far better adsorption properties [52]. This was a crucial improvement that also facilitated the development of the affinity-coating-based B_mem_ assays described here. Additionally, the introduction of automated, objective, and validated machine reading of T cell secretory footprints also contributed seminally to the success of T cell ImmunoSpot assays. In contrast to T cell assays, in which the spot morphologies follow simple rules (as the anti-cytokine-specific capture antibody’s affinity for the analyte is fixed [53] and therefore objective automated size gating can be applied [54,55]), for direct B cell assays (Figure 3A,B), the secretory footprints’ features are primarily defined by the affinity of the ASC-derived antibody for the membrane-bound antigen. Thus, the analysis of direct B cell ImmunoSpot assays needs to follow a different set of spot recognition rules [44]. Together with high dynamic range (HDR) imaging, this methodology allows high-content analysis of secretory footprints in B cell ELISPOT/FluoroSpot assays introducing new dimensions for the investigation of antigen-specific ASC repertoires. The introduction of the affinity coating approach (enabling rapid deployment of ImmunoSpot assays for more-or-less any desired antigen), along with the assay’s utilization for affinity and cross-reactivity studies as described above, together with suitable analytical software may finally allow the introduction of more widespread analysis of the antigen-specific B_mem_ repertoire, even meeting the strict demands of regulated and routine clinical monitoring applications.

As measurements of circulating antibodies in blood (plasma/serum) do not reveal many critical aspects of humoral immunity (as outlined above) the newly gained ability to readily monitor the antigen-specific B_mem_ compartment should provide much-needed insights into the mechanisms underlying humoral immune reactivity.

## Data Availability

The data presented in this manuscript will be made available by the authors, without undue reservations, to any qualified research.
